# The Effects of a Single Vagus Nerve’s Neurodynamics on Heart Rate Variability in Chronic Stress: A Randomized Controlled Trial

**DOI:** 10.3390/s24216874

**Published:** 2024-10-26

**Authors:** Ana Isabel Pérez-Alcalde, Fernando Galán-del-Río, Francisco J. Fernández-Rodríguez, Marta de la Plaza San Frutos, María García-Arrabé, María-José Giménez, Beatriz Ruiz-Ruiz

**Affiliations:** 1 Department of Physiotherapy, Faculty of Sport Sciences, Universidad Europea de Madrid, 28670 Madrid, Spain; anaisabel.perez@universidadeuropea.es (A.I.P.-A.); francisco.fernandez@universidadeuropea.es (F.J.F.-R.); marta.delaplaza@universidadeuropea.es (M.d.l.P.S.F.); maria.gararrabe@universidadeuropea.es (M.G.-A.); beatriz.ruiz@universidadeuropea.es (B.R.-R.); 2 International Doctoral School, Rey Juan Carlos University, 28008 Madrid, Spain; 3 Department of Physical Therapy, Occupational Therapy, Rehabilitation and Physical Medicine, Faculty of Health Sciences, Rey Juan Carlos University, 28922 Madrid, Spain; fernando.galandel@urjc.es

**Keywords:** neurodynamics, parasympathetic activation, heart rate variability, chronic stress, autonomic nervous system, heart reliability variability metrics, vagus nerve

## Abstract

Background: The modulation of the autonomic nervous system’s activity, particularly increasing its parasympathetic tone, is of significant interest in clinical physiotherapy due to its potential benefits for stress-related conditions and recovery processes. This study evaluated the effectiveness of the addition of neurodynamics in enhancing parasympathetic activation in subjects with chronic stress. Methods: A clinical trial randomly assigned participants to a group with neurodynamics (6 bpm breathing protocol + manual therapy + neurodynamic technique) or a group without neurodynamics (6 bpm breathing protocol + manual therapy only). Metrics of heart rate variability (HRV), including the Mean Heart Rate (Mean HR), standard deviation of intervals between consecutive heartbeats (SDNN), Heart Rate Difference (Diff. HR), Root Mean Square of Successive Differences (RMSSD), number of intervals differing by more than 50 ms (NN50), percentage of consecutive NN intervals that differed by more than 50 ms (pNN50), and the high-frequency component measured in standardized units (HF), were assessed before, during, and after the intervention. Results: During the intervention, the group with neurodynamics showed significant changes in all variables except in the pNN50 and HF while the group without neurodynamics only showed improvements in the Mean HR, SDNN, and RMSSD. In the post-intervention phase, the group with neurodynamics maintained an increase in HRV while the group without neurodynamics experienced a decrease, suggesting an increase in sympathetic activity. Conclusions: Vagal nerve neurodynamics appear to represent an effective method for enhancing parasympathetic activation in patients with chronic stress. The results highlight the importance of a more comprehensive analysis of HRV variables in order to obtain a correct picture of the impact of interventions on the complex and multifaceted functioning of the autonomic nervous system.

## 1. Introduction

The balance between the sympathetic nervous system (SNS) and the parasympathetic nervous system (PNS) is essential for regulating the body’s physiological processes and maintaining homeostasis [1], allowing the body to cope with external influences, through feedback and self-regulation mechanisms [2].

The SNS prepares the body to respond to adverse situations through the “fight or flight” response [3], including, among others, increases in heart and respiratory rates, blood pressure, pupil dilation, glucose release, sweating, the production of stress hormones such as cortisol and catecholamines, and the redirection of blood flow to skeletal muscles [4]. On the other hand, the vagus nerve is the main representative of the PNS [5], and its functions are opposite to those of the SNS, promoting relaxation and repair [6,7].

Stress is a significant problem in today’s society, affecting a considerable part of the population. The World Health Organization estimates that more than 11 million people worldwide suffer from it [8]. According to the Gallup Global Emotions 2023 report [9], 40% of adults surveyed worldwide had experienced a lot of stress the day before the survey.

Chronic stress, characterized by the constant activation of acute stress circuits even when the original cause of the stress has disappeared [10], causes continuous increases in cortisol, adrenaline, noradrenaline, and norepinephrine [11] levels, as well as alterations in dopamine [12], serotonin [13], and glutamate levels [14,15]. These changes lead to structural modifications in the brain, including increases in the sizes of the amygdala and prefrontal cortex and a decrease in the size of the hypothalamus [16], key structures in emotional control, memory, and decision making [17]. In addition, chronic stress affects the gut–brain axis [18] and causes epigenetic changes [19,20]. The experience of chronic stress may be related with cardiovascular diseases [4], gastrointestinal problems [21], sleep disorders [22], metabolic disorders [1], and anxiety and depression [23,24,25] among other problems.

Perceived stress refers to a person’s cognitive evaluation of the magnitude of threat posed by stressors and his or her ability to cope with them [25]. During this process, it is common for individuals to experience negative emotions such as anxiety, depression, anger, and grief [26].

One of the physiological processes regulated by the autonomous nervous system (ANS) is heart rate variability (HRV) [27], which is defined as a measure that captures fluctuations in the intervals between consecutive heartbeats, known as the R-R (RR) or N-N (NN) interval when interference is removed [28]. HRV can be analyzed using different approaches, including time domain, frequency domain, and non-linear analysis methods [29].

Several key parameters are included in the time domain. The Mean HR is the average of all recorded heart rate intervals [30]; the RMSSD reflects the variability in intervals between adjacent beats and is closely related to parasympathetic activity [31]. The SDNN measures the standard deviation of interbeat intervals and provides an overview of the total variability, influenced by both branches of the ANS [32]. Other parameters in this area are the NN50 and pNN50, wherein higher values indicate better autonomic regulation [33]. In addition, Diff HR provides a measure of the amplitude of HR variation [27].

Frequency domain measurements allow the HRV signal to be decomposed into different frequency components. The High-Frequency Component (HF) is closely associated with parasympathetic or vagal activity and is related to physiological processes such as breathing and relaxation [29]. On the other hand, the Low-Frequency Component (LF) reflects both sympathetic and parasympathetic influences [34]. The LF can be used in both short and long recordings. However, its interpretation in short recordings should be performed with caution as the influences of both nervous systems can vary significantly over short periods of time [27]. In addition to these bands, the LF/HF ratio is used to assess the balance between sympathetic and parasympathetic activities [35], although studies such as Billman’s have questioned this claim [36].

Additionally, Ultra-Low-Frequency (ULF) and Very-Low-Frequency (VLF) bands, which reflect long-term factors like circadian rhythms and metabolic processes, require extended recordings (24 h) [29,32].

In addition, HRV can be analyzed by non-linear methods, such as approximate entropy analysis and trendless fluctuation analysis, which quantify short-term variability [30].

Slow breathing may influence the oscillations of HRV and blood pressure due to the stimulation of aortic arch baroreceptors [37]. To prevent breathing from being a confounding factor in HRV assessment, some studies have suggested the use of consciously controlled breathing to standardize the method [38].

To cope with chronic stress, it is crucial to find therapeutic tools that support PNS activity, counteracting the negative effects of the constant increases in cortisol and catecholamines produced by the SNS [39]. Therapeutic tools that have been tested include meditation [40], acupuncture [41], aromatherapy [42], deep diaphragmatic breathing [43], nature walks [44], forest baths [45], singing exercises [46], physical exercise [47,48], and vagus nerve neurostimulation [49].

Deep diaphragmatic breathing, performed at a rate of six breaths per minute (6 bpm), has been extensively researched and is considered the gold standard for achieving greater parasympathetic activation [43,50,51].

Neurodynamics is a physiotherapy technique that improves the functionality of nerves affected by nerve entrapment along their pathway [52,53,54]. Its impact on the vagus nerve could influence its functionality and thus HRV [55]. From an anatomical and functional perspective, the vagus nerve is crucial to the physiology of the PNS, extending from the brainstem to the abdomen and innervating multiple vital organs [56]. To our knowledge, there had been only one previous study in which neurodynamics were applied to the vagus nerve, with favorable results in modulating HRV in healthy subjects [55]. However, the impact on chronically stressed individuals remained to be studied.

Therefore, the aim of the present study was to investigate the effects on HRV of the inclusion of the neurodynamic technique in a 6 bpm guided breathing protocol associated with manual therapy.

## 2. Materials and Methods

### 2.1. The Study Design

A randomized experimental clinical trial was designed with blinding of the investigator performing the data analysis. The study was conducted in accordance with the Helsinki principles of the World Medical Association for clinical research involving human subjects. The Ethics Committee of Hospital Clínico San Carlos (Madrid, Spain) approved the study protocol (code 23/522-EC_X_Thesis). In addition, this clinical trial was registered in ClinicalTrials.gov (accessed on 12 July 2024) with the code NCT06499662.

### 2.2. Study Subjects

Participants were recruited according to inclusion and exclusion criteria from among employees of the European University of Madrid, Spain, from January to March 2024 using informational posters appropriately distributed throughout the facilities. Inclusion criteria included individuals over 18 years of age with self-perceived stress of at least six months’ duration. Exclusion criteria were as follows: consumption of tea, caffeine, energy drinks, alcohol or tobacco in the two hours prior to the study; neck pain or significant headache; carotid sinus syndrome; pregnancy; recent cervical or cardiac surgery; recent significant trauma; cancer; neurological disorders affecting muscle tone; underlying diseases such as diabetes mellitus or hypertension; beta-blocker use; and arrhythmias or other cardiac diseases [55].

### 2.3. Intervention Protocol

**Figure 1:**
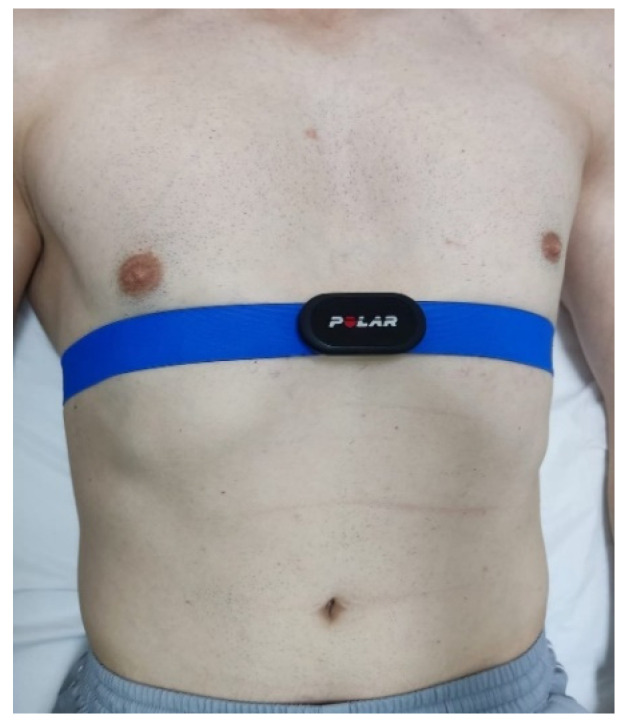
HRV monitoring via the Polar^®^ H10 band (Polar Electro Kempele, Finland).

Each participant was placed supine on a stretcher with a cushion under their knees in a room with an ambient temperature of 22 °C and indirect lighting. After five minutes, the estimated time to stabilize vital signs, a Polar H10 band (Polar Electro, Kempele, Finland) impregnated with conductive gel was placed on the chest, just below the submammary fold, so that the sensor was positioned over the sternum (Figure 1). Once the band was in place, the pre-intervention HRV measurement began, recording HRV values for 7 min while the person was at rest and breathing freely. Subsequently, each participant in both groups started the intervention.

Group with neurodynamics: Patients followed the 6 bpm breathing protocol associated with manual therapy combined with the neurodynamic technique. 

Group without neurodynamics: Patients followed the 6 bpm breathing protocol associated with manual therapy only.

#### 2.3.1. Breathing Protocol Associated with Manual Therapy

Patients maintained guided deep abdominal breathing at 6 bpm for 15 min, inhaling and exhaling through the nose. This baseline breathing at 6 bpm was chosen based on the fact that cardiac coherence is a state in which the heart rate follows a regular and harmonic pattern, improving the function of the autonomic nervous system (ANS) [57]. This state is achieved by breathing at 6 bpm, coinciding with the resonant frequency of the cardiovascular system, which amplifies heart rate variability (HRV) [58]. As a result, baroreflex function is optimized, blood pressure is reduced, and synchronization between the respiratory and cardiovascular systems is improved, increasing stress resistance [59]. In addition, respiratory sinus arrhythmia (RSA), a natural variation of the heart rate that is synchronized with breathing, is also amplified with this slow and regular breathing, further improving autonomic regulation and HRV [60].

Breathing was guided by a recording in which a gong marked the start of each 5 s inspiration and another gong marked the start of the 5 s exhalation. The person in charge of the intervention constantly monitored that the breathing was diaphragmatic and in rhythm with the sounds of the recording.

During the first 3 min, a manual suboccipital inhibition technique was associated with breathing. The physiotherapist, positioned at the head of the couch, placed the pads of all eight fingers along the suboccipital line until tissue relaxation was noted [61].

#### 2.3.2. Neurodynamic Technique

**Figure 2:**
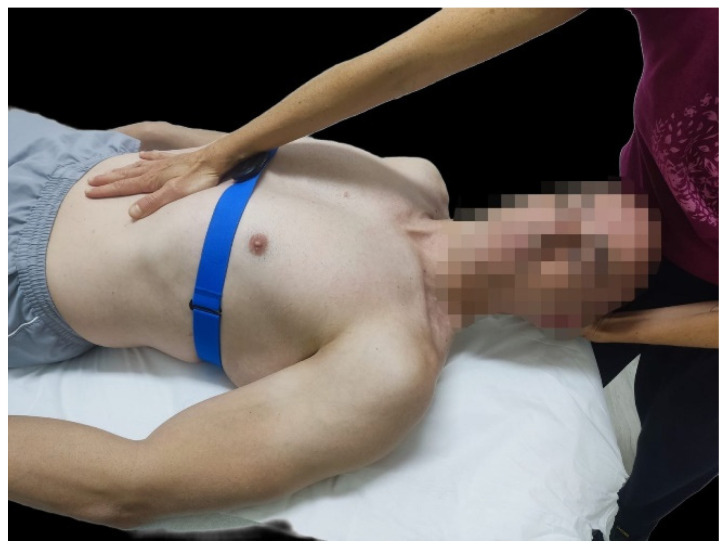
Neurodynamic technique used with the vagus nerve.

The neurodynamic technique consisted of passively introducing movements in the cervical region: +/−11° of rotation homolateral to the vagus nerve to be treated, +/−8.5° of contralateral lateral flexion, and +/−8.5° of flexion (Figure 2). These parameters were chosen following the study by Carta G et al., which had evaluated the reliability of this technique in healthy subjects [55]. This maneuver was applied for 6 min on the right vagus nerve and for another 6 min on the left vagus nerve. The procedure was performed only once for both groups, with no additional training or repeated trials; it used a single observation to assess the immediate effects of the intervention.

For conducting the technique on the right vagus nerve, the physiotherapist placed the palm of her left hand under the occipital nerve, grasping the right occipital condyle with the index and middle fingers, and introduced the above-mentioned parameters during exhalation. Simultaneously, the physiotherapist exerted a depression in the upper abdomen with the heel of her right hand, applying gentle pressure dorsally and caudally, in rhythm with the person’s exhalation (Figure 2). During inspiration, both hands progressively reduced the tension to the initial resting position. This technique was replicated in the same way for the left vagus nerve.

**Figure 3:**
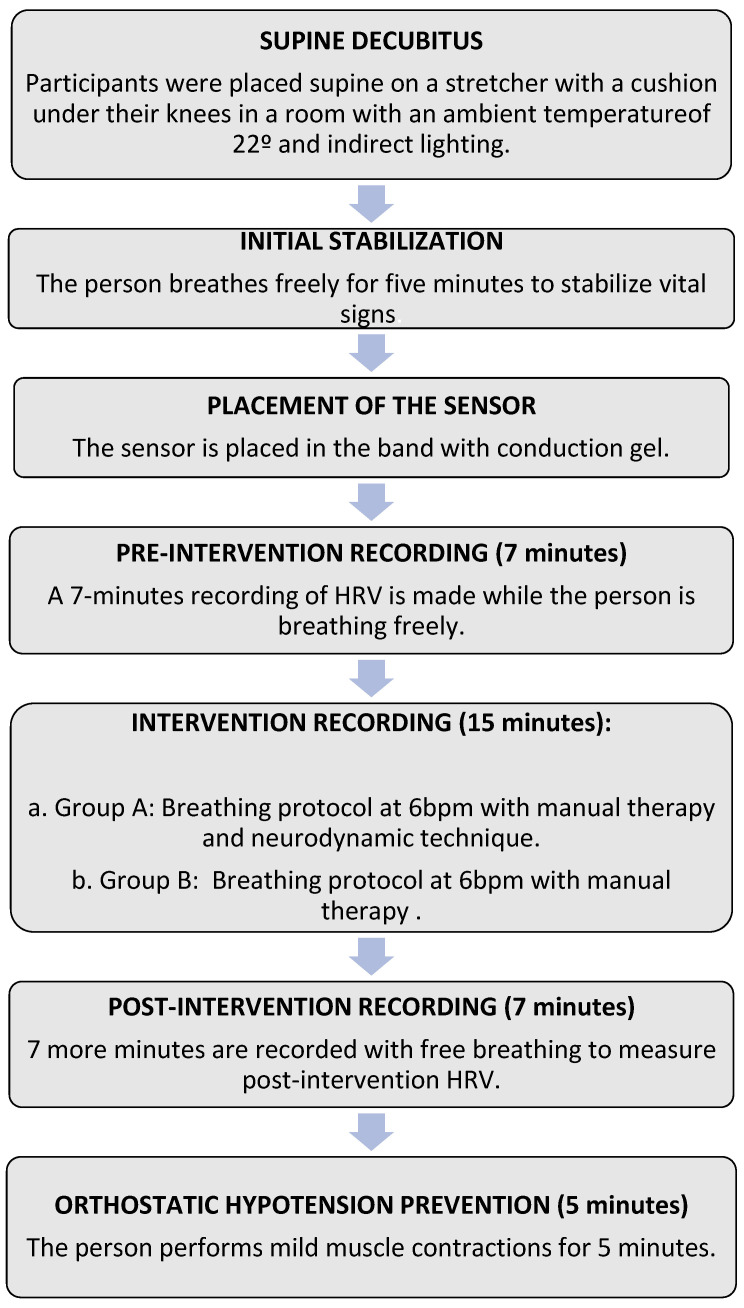
Real-time diagram of the experiment.

The duration of the intervention for each of both groups was 15 min, and during the whole time, the patients were monitored with the Polar H10 band to record the HRV during the intervention. After the intervention, the subjects remained on the stretcher for another 7 min breathing freely to record post-intervention HRV and for additional 5 min while performing gentle muscle contractions to prevent orthostatic hypostatism [62,63]. In Figure 3, the chronological order of the entire process is shown.

### 2.4. Data Analysis Procedure

For data analysis, short-term (5 min of each recording taken) HRV recordings were used, following the guidelines established by the Task Force of the European Society of Cardiology and the North American Society of Pacing and Electrophysiology [64], at three different time points: pre-intervention, during intervention, and immediately post intervention. Subsequently, the recordings were processed using Kubios HRV Scientific Lite 4.1.0 software (Kubios Oy, Kuopio, Finland).

HRV recordings were thoroughly reviewed, and any errors or anomalies were corrected following rigorous criteria, ensuring a corrected artefact rate of less than 0.001%, as recommended in the scientific literature [30].

Data recorded consisted of Mean HR [30], RMSSD [31], SDNN [32], NN50 [33], pNN50 [33], Diff HR [27], and HF measured in standardized units (n.u.) [29].

### 2.5. Statistical Analysis

The sample size was calculated using Granmo 8.0 software, using HRV as the main variable, according to data provided by Hernando et al. [65]. An alpha risk of 0.05 and a power of 0.8 in a bilateral contrast were established, determining the need for 31 subjects per group to detect a difference of 0.12 in HRV, assuming a common standard deviation of 0.15. In addition, a loss-to-follow-up rate of 20% was considered.

Statistical analysis was performed using Jamovi software (version 2.3.28). Qualitative variables were described by absolute and relative frequency, and for quantitative variables, the median and interquartile range (P25, P75) were used given the dispersion of the data. Before proceeding with the inferential analyses, the normality of the variables was assessed using the Shapiro–Wilk test and Q-Q plots. Given the non-normal nature of the distribution of the variables, the analysis was performed using non-parametric tests; specifically, the Mann–Whitney U test was used to compare two independent groups and the Wilcoxon test was used for paired groups. The results were considered significant with a *p*-value ≤ 0.05.

## 3. Results

**Figure 4:**
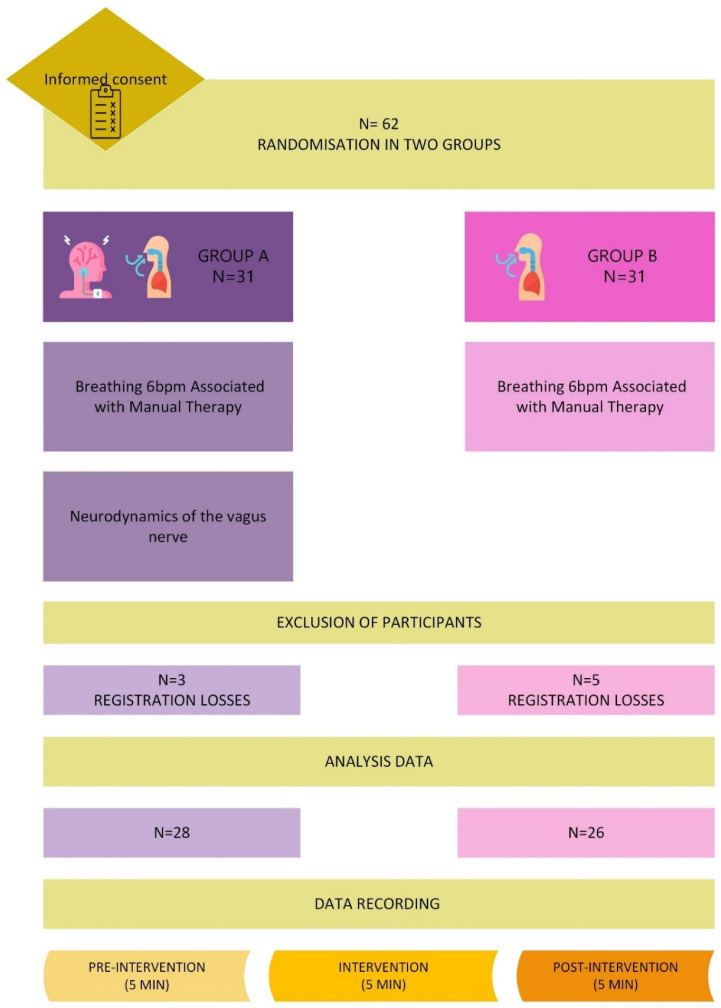
Flowchart detailing the study processes.

Sixty-two individuals were included, of which eight were then excluded due to errors in the records, so the data analysis ultimately included 54 individuals, 28 subjects in the group with neurodynamics and 26 subjects in the group without neurodynamics. Figure 4 shows the study flowchart detailing the study process and the recruited participants.

Non-statistically significant differences were found when comparing the demographic and anthropometric characteristics of participants between groups as shown in Table 1.

Table 2 shows comparisons of HRV variables between study groups before, during, and after the intervention. Except in the case of the HF variable during the intervention, no significant differences were observed with the addition of the neurodynamic technique to the breathing protocol.

Table 3 shows the comparison within study groups of HRV variables between the pre-intervention and during-intervention stages, as well as between the pre-intervention and post-intervention stages.

During the intervention, in the group with neurodynamics, the values of the SDNN, Diff HR, RMSSD, and NN50 were significantly higher than those pre-intervention; in contrast, the values of the Mean HR and HF band were significantly lower. Changes showed similar trends in the group without neurodynamics, but statistical differences were only observed for the Mean HR, SDNN, RMSSD, and HF.

Post intervention, in the group with neurodynamics, only the values of the Mean HR were significantly lower than those pre-intervention. In the group without neurodynamics, changes were more pronounced, with statistically significant reductions in the Mean HR, NN50, and pNN50 values.

Table 4 shows the differences between pre-intervention and during-intervention values, as well as between pre-intervention and post-intervention values by study group, and the between-groups comparisons.

No significant differences in the magnitude of mean pre-intervention and during-intervention difference values or mean pre-intervention and post-intervention difference values were found between the study groups.

## 4. Discussion

The present study investigated the effects of the inclusion of the neurodynamic technique in a 6 bpm guided breathing protocol associated with manual therapy in chronically stressed individuals to assess the impact on HRV of neurodynamics applied to the vagus nerve. Although no significant differences were found when comparing both groups, intragroup comparisons showed significant improvements in several HRV parameters during the intervention, being more pronounced in the group receiving the neurodynamic technique. Changes were also more prolonged in the group with neurodynamics than in the group without it, where significant changes in the opposite direction were observed.

The lying position during the experiment and a baseline breathing rate of 6 bpm were chosen for both groups in order to standardize the sample and to avoid fluctuations in the results depending on the type of breathing performed by each participant [38]. This standardization made possible to specifically assess the effect of neurodynamics on HRV based on the assumption that breathing at 6 bpm would induce parasympathicotonia in all participants.

In the data recorded during the intervention, changes in the group with neurodynamics (lower values of the Mean HR and higher values of the SDNNN, DifHR, RMSSD, and NN50 versus pre-intervention) indicated greater parasympathetic activation. In the group without neurodynamics, parasympathetic activation was also observed, but changes were only significant for three parameters: the Mean HR, SDNN, and RMSSD.

In the post-intervention phase, the group with neurodynamics maintained the significant decrease in the Mean HR, reflecting a sustained increase in HRV. On the other hand, the group without neurodynamics maintained also the significant decrease in the Mean HR but with significant decreases in the RMSSD, NN50, and pNN50, showing increased sympathetic activity. This increase in the SNS activity contrasted with data in the literature that considered breathing at 6 bpm the gold standard for increasing HRV [43,50,51].

Therefore, although no significant differences were observed between the groups, participants in the group with neurodynamics showed a tendency to increase HRV both during and after the intervention. In contrast, participants in the group without neurodynamics exhibited a tendency to decrease HRV in the post-intervention phase. This suggests that although breathing at 6 bpm modulates ANS activity while it is practiced, once it is ceased, an increase in sympathetic activity is observed when the neurodynamic technique is not applied. In addition, our findings also provide new evidence for the effectiveness of neurodynamics in modulating the ANS and HRV in chronically stressed subjects, proposing a viable alternative to improve autonomic health in the short term.

Our results contrast with previous studies that advocated breathing at 6 bpm as the type of breathing that produced the greatest increase in HRV. This discrepancy may be due to methodological differences. Many previous studies used a limited number of HRV variables and may have omitted important aspects of cardiac and autonomic dynamics, leading to incomplete conclusions about the complex and multifaceted functioning of the ANS [66,67,68,69,70,71,72]. Bonechere et al. [66] based their conclusions on a significant increase in the LF band as being the sole parameter measured. Some studies suggested that the LF band reflects a complex interaction between the SNS and SNP, especially during slow respiratory rhythms, and that thus, the increase in the LF could not be considered a good indicator of sympathetic activation [32,73,74]. Magnon et al. [67] based their conclusions on HRV solely on the HF value during 6 bpm breathing, a parameter that is well known to be influenced by breathing and, therefore, would not, on its own, be indicative of what is happening with HRV [36,74]. Other studies [68] focused primarily on the RMSSD and HF, omitting other metrics, which may have limited their ability to detect changes in sympathetic activity. The study by Steffen et al. [69], on the other hand, based its conclusions on the analysis of the LF/HF ratio, although it is known that this ratio alone cannot provide accurate information on sympathovagal balance [32,36].

Likewise, the study by Linl et al. [70] reported significant results only for the SDNN and LF, and the study by Wu et al. [71], although including the LF/HF ratio, did not obtain statistically significant results for the HF band. Soer et al. [72] evaluated the efficacy of slow breathing at different bpm values (4, 5, and 7 bpm) and reported results on different variables (SDNN at 5 bpm, RMSSD at 7 bpm, and HF at 4 bpm), which provided different information.

In line with our results, You et al. [75] explained that contrary to their initial hypothesis, the intensity of perceived stress and emotional arousal were found to increase after a slow breathing exercise.

Regarding the types of subjects commonly used in studies validating 6 bpm breathing as a technique capable of improving HRV, it is important to note that some studies [67,69,72,76,77] included healthy subjects and extrapolated their findings to the management of both acute and chronic stress. However, this extrapolation might not always be adequate because of the physiological adaptations that occur in chronically stressed individuals. In situations of chronic stress, the ANS may undergo dysregulations that significantly alter the HRV response, differing from the response observed in healthy subjects. In this sense, significant differences in ANS responses have been highlighted between healthy and chronically stressed subjects [3,29].

The subject’s position and the study context may also affect the results. In our study, the subjects were kept in a supine position without engaging in any additional activity. It has been reported that HRV is highly sensitive to changes in position [30,77] and sex [78]. The lack of stability in HRV during rest questions the current methodologies and confirms the need for standardized protocols for comparison between studies following the recommendations of the Task Force of the European Society of Cardiology and the North American Society of Pacing and Electrophysiology [64].

### 4.1. Study Limitations

One limitation of our study was the failure to account for participants’ prior training in slow breathing. A lack of familiarity with the breathing technique could have contributed to a sympathetic response due to the novelty and potential initial discomfort. This factor may have affected the stability of HRV measurements, limiting the ability to generalize the results.

Another limitation was the absence of long-term measurements (1 h/1 day after the intervention) to observe whether HRV values underwent changes once they stabilized after the intervention.

It would have been interesting to include some type of questionnaire or scale to measure relaxation or anxiety before and after the interventions as a complement to the parameters measured in this study as had been used in a previous study [70].

### 4.2. Future Research

Future research should focus on evaluating the impact of prior training in slow breathing for participants, which could help determine whether familiarity with the technique alters the results obtained. Additionally, investigations considering variability in body positions and activities during interventions would help us better understand how these factors affect HRV. It would also be beneficial to explore the effect of neurodynamics in different populations, including healthy individuals and those without baseline breathing patterns, to better generalize the findings.

Furthermore, it would be necessary to reassess the effectiveness of current therapeutic recommendations (6 bpm breathing) for individuals with chronic stress.

### 4.3. Clinical Applications

In clinical physiotherapy practice, inducing an increase in the parasympathetic tone in patients is of considerable interest [74] as it can both enhance regeneration and recovery processes as well as reduce inflammation associated with stress [74,78]. The activation of the PNS decreases the inflammatory response and promotes a state of relaxation and tissue repair [79], potentially boosting the effectiveness of physiotherapeutic treatments. Therefore, optimizing parasympathetic tone through vagus nerve mobilization using neurodynamic techniques prior to manual intervention in patients with chronic stress could significantly enhance therapeutic effectiveness, offering faster and more sustained improvement.

## 5. Conclusions

Our findings provide new evidence that the addition of neurodynamics may be an effective strategy for maintaining increased parasympathetic activation. The results highlight the importance of a more comprehensive analysis of HRV variables in order to obtain a correct picture of the impacts of interventions on the complex and multifaceted functioning of the ANS. Future studies are warranted to further investigate the benefits of neurodynamics on the modulation of the ANS.

## Figures and Tables

**Table 1:** Demographic and anthropometric characteristics of the participants by study group.

Variable	Total (n = 54)	Group with Neurodynamics (n = 28)	Group Without Neurodynamics (n = 26)	*p*
Age (years)	45.5 (40.0, 52.0)	44.5 (35.5, 52.3)	46.5 (42.3, 52.0)	0.204
Sex (Male, n (%))	13 (24.1)	9 (32.1)	4 (15.4)	0.150
Height (cm)	167.0 (160.0, 170.0)	165.0 (160.8, 169.3)	167.0 (160.0, 170.0)	0.975
Weight (kg)	67.0 (60.5, 75.0)	70.0 (61.5, 75.5)	65.0 (60.5, 73.3)	0.415
BMI (kg/m2)	24.1 (22.9, 26.0)	24.1 (22.8, 26.1)	24.1 (22.9, 25.9)	0.556

Data are presented as median (interquartile range) values except where indicated.

**Table 2:** Comparison of HRV variables between study groups before, during, and after the intervention.

Variable	Total (n = 54)	Group with Neurodynamics (n = 28)	Group Without Neurodynamics (n = 26)	*p*
**Pre-intervention**				
Mean HR (bpm)	66.3 [60.5, 71.5]	64.7 [60.3, 70.2]	67.0 [62.5, 76.7]	0.497
SDNN (ms)	34.6 [25.1, 48.1]	33.3 [25.4, 46.2]	36.2 [25.1, 52.7]	0.455
Diff HR (bpm)	15.1 [11.4, 18.1]	14.6 [10.9, 17.6]	15.8 [12.7, 18.1]	0.508
RMSSD (ms)	31.9 [21.1, 45.6]	30.7 [20.3, 44.4]	33.5 [22.9, 47.6]	0.790
NN50 (number of NN intervals)	23.0 [7.0, 73.3]	22.0 [7.0, 75.3]	33.5 [10.3, 69.8]	0.723
pNN50 (%)	0.07 [0.02, 0.24]	0.07 [0.02, 0.23]	0.09 [0.03, 0.24]	0.684
HF (n.u.)	35.0 [18.6, 53.7]	35.4 [17.1, 54.1]	34.8 [24.7, 52.7]	0.911
**During intervention**				
Mean HR (bpm)	64.0 [58.6, 71.5]	61.4 [59.5, 66.1]	66.5 [56.8, 73.1]	0.444
SDNN (ms)	50.5 [39.5, 71.6]	50.5 [40.6, 67.5]	51.2 [36.2, 76.1]	0.843
Diff HR (bpm)	16.5 [12.5, 21.1]	14.8 [12.1, 21.7]	17.0 [13.7, 19.9]	0.665
RMSSD (ms)	36.6 [25.1, 50.9]	38.6 [29.0, 50.7]	36.2 [21.4, 61.1]	0.519
NN50 (number of NN intervals)	48.0 [18.3, 90.0]	49.0 [24.3, 81.8]	47.0 [12.0, 94.3]	0.562
pNN50 (%)	0.15 [0.06, 0.32]	0.15 [0.07, 0.29]	0.16 [0.04, 0.33]	0.917
HF (n.u.)	12.1 [8.1, 19.4]	14.3 [10.1, 20.4]	9.2 [6.8, 13.2]	**0.012**
**Post intervention**				
Mean HR (bpm)	63.1 [57.7, 71.9]	62.7 [57.3, 71.7]	67.3 [59.1, 72.3]	0.519
SDNN (ms)	32.3 [24.7, 41.9]	32.3 [23.6, 44.1]	32.4 [26.7, 41.7]	0.712
Diff HR (bpm)	13.3 [10.2, 18.7]	12.6 [9.8, 18.8]	13.6 [12.3, 18.5]	0.260
RMSSD (ms)	29.3 [20.0, 39.2]	28.6 [19.9, 39.4]	29.3 [21.0, 37.9]	0.938
NN50 (number of NN intervals)	29.5 [9.0, 48.0]	22.5 [6.0, 52.3]	32.0 [9.0, 43.3]	0.703
pNN50 (%)	0.08 [0.02, 0.16]	0.06 [0.02, 0.20]	0.08 [0.03, 0.13]	0.710
HF (n.u.)	35.3 [25.4, 49.0]	34.2 [26.2, 55.4]	36.2 [24.1, 46.3]	0.764

Data are presented as median [interquartile range] values. Abbreviations: Mean Heart Rate (Mean HR), Standard Deviation of NN Intervals (SDNN), Heart Rate Difference (Diff HR), Root Mean Square of Successive Differences (RMSSD), Number of NN50 Intervals (NN50), Percentage of NN50 Intervals (pNN50), Normalized High-Frequency Component (HF), bpm (beats per minute), ms (milliseconds), % (percentage), and n.u. (normalized units).

**Table 3:** Comparison within study groups of HRV variables in pre-intervention versus intervention stages and pre-intervention versus post-intervention stages.

**Pre-Intervention vs. Intervention**		
Variable	Group with neurodynamics (n = 28)	Group without neurodynamics (n = 26)
Mean HR (bpm)	0.002	<0.001
SDNN (ms)	<0.001	<0.001
Diff HR (bpm)	0.032	0.380
RMSSD (ms)	<0.001	0.049
NN50 (number of NN intervals)	0.004	0.100
pNN50 (%)	0.572	0.080
HF (n.u.)	<0.001	<0.001
**Pre-Intervention vs. Post Intervention**		
Variable	Group with neurodynamics (n = 28)	Group without neurodynamics (n = 26)
Mean HR (bpm)	0.010	0.024
SDNN (ms)	0.537	0.105
Diff HR (bpm)	0.662	0.437
RMSSD (ms)	0.274	0.053
NN50 (number of NN intervals)	0.263	0.018
pNN50 (%)	0.572	0.041
HF (n.u.)	0.762	0.499

Abbreviations: Mean Heart Rate (Mean HR), Standard Deviation of NN Intervals (SDNN), Heart Rate Difference (Diff HR), Root Mean Square of Successive Differences (RMSSD), Number of NN50 Intervals (NN50), Percentage of NN50 Intervals (pNN50), Normalized High-Frequency Component (HF), bpm (beats per minute), ms (milliseconds), % (percentage), and n.u. (normalized units).

**Table 4:** Differences between pre-intervention and during-intervention values and between pre-intervention and post-intervention values by study group.

Variable	Group with Neurodynamics (n = 28)	Group Without Neurodynamics (n = 26)	*p*
**Pre-intervention—during intervention**			
Mean HR (bpm)	2.48 [0.92, 4.04]	3.05 [1.74, 4.35]	0.661
SDNN (ms)	−23.30 [−31.70, −15.00]	−16.70 [−22.50, −10.90]	0.322
Diff HR (bpm)	−1.56 [−2.99, −0.13]	−1.12 [−3.22, 0.97]	0.600
RMSSD (ms)	−11.50 [−18.30, −4.62]	−5.40 [−10.40, −0.43]	0.267
NN50 (number of NN intervals)	−20.10 [−32.20, −7.96]	−6.81 [−18.8, 5.14]	0.215
pNN50 (%)	−0.07 [−0.11, −0.03]	1.99 [−2.39, 6.36]	0.377
HF (n.u.)	22.10 [14.60, 29.60]	26.10 [17.30, 34.90]	0.465
**Pre-intervention—post intervention**			
Mean HR (bpm)	2.02 [0.34, 3.70]	1.79 [0.29, 3.29]	0.803
SDNN (ms)	0.57 [−4.05, 5.20]	3.64 [−0.77, 8.04]	0.404
Diff HR (bpm)	0.39 [−1.53, 2.31]	0.64 [−1.78, 3.05]	0.870
RMSSD (ms)	2.40 [−1.44, 6.25]	4.06 [0.37, 7.75]	0.486
NN50 (number of NN intervals)	8.07 [−2.51, 18.60]	13.90 [3.28, 24.60]	0.359
pNN50 (%)	0.02 [−0.02, 0.05]	2.16 [−2.21, 6.53]	0.245
HF (n.u.)	−0.52 [−8.72, 7.68]	2.13 [−6.63, 10.9]	0.476

Data are expressed as mean difference [95% confidence interval]. Abbreviations: Mean Heart Rate (Mean HR), Standard Deviation of NN Intervals (SDNN), Heart Rate Difference (Diff HR), Root Mean Square of Successive Differences (RMSSD), Number of NN50 Intervals (NN50), Percentage of NN50 Intervals (pNN50), Normalized High-Frequency Component (HF), bpm (beats per minute), ms (milliseconds), % (percentage), and n.u. (normalized units).

## Data Availability

Datasets supporting the reported results are available upon request from the first author.

## Abbreviations

6 bpm	6 breaths per minute
AAQ-II	Acceptance and Action Questionnaire-II
ANS	Autonomic Nervous System
BMI	Body Mass Index
HR Diff	Difference between Maximum and Minimum Heart Rate
HF	High-Frequency Component
HRV	Heart Rate Variability
LF/HF	Ratio between the low- and high-frequency bands
LF	Low-Frequency Component
max HR	Maximum Heart Rate
Mean HR	Mean Heart Rate
min HR	Minimum Heart Rate
NN	Intervals between consecutive heartbeats when interference is removed
NN50	Number of NN Intervals differing by more than 50 ms
pNN50	Percentage of NN Intervals differing by more than 50 ms
PNS	Parasympathetic Nervous System
PSS	Perceived Stress Scale
RMSSD	Root Mean Square Difference of the Successive Differences
RR	Intervals between consecutive heartbeats
SDNN	Standard Deviation of NN Intervals
SNS	Sympathetic Nervous System
UCLA	University of California, Los Angeles (Loneliness Scale)
WHO	World Health Organization
